# Impact of work and coping factors on mental health: Australian truck drivers’ perspective

**DOI:** 10.1186/s12889-023-15877-4

**Published:** 2023-06-06

**Authors:** Elizabeth Pritchard, Caryn van Vreden, Ting Xia, Sharon Newnam, Alex Collie, Dan I. Lubman, Abilio de Almeida Neto, Ross Iles

**Affiliations:** 1 School of Public Health and Preventive Medicine, Monash University, Melbourne, VIC Australia; 2 Monash University Accident Research Centre, Melbourne, VIC Australia; 3 Turning Point, Eastern Health and Monash Addiction Research Centre, Eastern Health Clinical School, Monash University, Victoria, Australia; 4 Centre for Work Health and Safety, New South Wales Government, Australia

**Keywords:** Truck Drivers, Qualitative research, Mental health, Australia, Risk and supportive factors

## Abstract

**Introduction:**

Truck driving is one of the most common male occupations worldwide. Drivers endure long working hours, isolation, separation from family, compromised sleep, and face rigid regulatory requirements. Studies have documented the work factors contributing to poor health outcomes, however these have not been explored in the Australian context. The aim of this grounded theory study was to explore the impact of work and coping factors on mental health of Australian truck drivers from their perspective.

**Methods:**

Recruitment used a purposive snowball sampling, through social media campaigns and direct email invites. Interview data were collected via phone/teleconference, audio recorded and typed verbatim. Inductive coding and thematic analysis were completed with triangulation of themes.

**Results:**

Seventeen interviews were completed (94% male). Six themes arose, two supporting (Connections; Coping methods), and four disrupting mental health (Compromised supports; Unrealistic demands; Financial pressures; Lack of respect). Drivers had concerns regarding the many things beyond their control and the interactions of themes impacting their health even further.

**Conclusion:**

This study explored the impact of work and coping factors affecting truck driver mental health in Australia. Themes described the importance of *connections* and *coping methods* drivers had to support their health. Many factors that compromised their health were often outside their control. These results highlight the need for a multi-faceted collaboration between stakeholders; the driver, employing companies, policy makers/regulators and the public to address the negative impact of truck driving on mental health.

**Supplementary Information:**

The online version contains supplementary material available at 10.1186/s12889-023-15877-4.

## Introduction

Truck driving is the most common vocation among men, with over 3 million drivers in Europe [1], > 2.7 million in the U.S. [2], and > 200,000 in Australia accounting for ~ 2% of the national labour force [3]. Truck driving exemplifies a sedentary occupation where chronic health conditions are highly prevalent with increased likelihood of cardiovascular disease, type 2 diabetes, musculoskeletal problems, isolation, stress, and fatigue [4, 5]. Findings from cross-sectional and cohort studies report these health conditions are further intensified with long working hours, lack of control over schedules, shift-work, social isolation, poor nutrition, reduced opportunity for physical activity, and fatigue, all of which negatively impact family life and relationships [4, 6–9]. Truck drivers experience all of these occupational factors and are therefore at increased risk of poorer health and work-related injury or illness, than other occupational groups [6].

The burden of chronic disease and mental ill-health on truck drivers is increasing worldwide, with health service, personal and social costs annually estimated at over $200 billion globally [10–12]. In Australia alone, over 120,000 compensation claims were accepted from truck drivers only for work-related injury or illness between 2004 and 2015, resulting in more than 22,317 working years of lost productivity (2,029 per annum) [13]. This is comparable to the entire Education and Training Sector of 2,207 full time equivalent years of time lost per annum, and approximately one third of years lost from the whole of the Health Care and Social Assistance industry (6,306), Manufacturing (5,083), and around half of years lost for construction (4,975) based on workplace injury and illness claims [14]. A recent publication using dynamic life table modelling of Australian male truck drivers over a 10-year period (data from Driving Health Project [13]) estimated projected healthcare costs could exceed $485 million (by 2030), costing AU$2.6 billion in lost productivity and AU$4.7 billion lost life-years [15]. The statistics and cost projections suggest that the impact of work and coping factors is extremely costly, highlighting the need for targeted interventions across the driving industry. Key areas of concern in the industry include lack of social support and occupational factors.

We know that family and social relationships are vital for mental health and psychological wellbeing [16, 17]. We also know that positive relationships have a beneficial effect on sleep and safety decision-making for drivers [18]. Social support is a significant factor in injury recovery for all workers [19] and receiving support from friends is also associated with lower usage of healthcare services [20]. The role of social support is important to consider in the context of heavy vehicle driving given the on-the-job isolation for truck drivers (especially long-haul > 500 kms per day) [17] and time away from family. In this context, it is understood that it is difficult for drivers to maintain effective relationships [20], which is a key consideration in developing driver health interventions.

A growing amount of qualitative research exploring the impact of occupational factors on health within the transport industry is emerging. A recent Canadian phenomenological study investigating long-haul truck driver health and healthcare experiences reported they felt dehumanized and isolated which challenged their ability to be connected and form protective relationships with others, including their healthcare providers [21]. A United States phenomenological study explored health-supportive behaviours of long-haul truck drivers to discover how they exhibit resiliency [22]. That study identified a broad range of factors affected health outcomes including level of access to resources, perceived and physical barriers (e.g. sedentariness and inaccessibility to healthy food), environmental settings (e.g. on the road gyms), to name a few. It also suggested that a holistic approach of “applying ecological theories of health behaviour and settings approaches” would be the most beneficial for improving truck drivers’ health [22]. A Canadian study (n = 16 drivers, n = 10 managers) investigating lifestyle issues and disease risk factors perceived by truck drivers and managers, reported stress (e.g. traffic, level of respect), lifestyle (e.g. nutrition, physical activity), culture (e.g. independence, taking pride in the job), family dynamics (e.g. missed events, guilt), and fatigue (e.g. irregular hours, sleep policy) as contributors of risks to health [23]. Another conducted in Iran (n = 18), explored truck drivers’ understanding and experiences of traffic crashes [24]. The study identified the lack of driver ability to control stress alongside multi-complex workplace factors beyond their control (e.g., poor scheduling, lack of proper equipment/facilities, and unsupportive work environments) were contributors to poorer health outcomes [24]. Similarities exist between these studies of recognising that there are multiple factors involved, many of which are outside of the driver’s control. However, much less is known about Australian truck drivers.

A recent Australian study interviewed injured workers from many occupations across three injury compensation schemes and their family members to explore factors impacting injury recovery through a qualitative and systems mapping approach [25]. That study demonstrated the complexity of the recovery process and acknowledged how changes occurring ‘upstream’ (at policy/regulatory levels) can positively impact injury recovery outcomes. It also highlighted the need to look beyond the ‘worker’ themselves for solutions.

Surveys conducted with Australian truck drivers as part of the Driving Health Project [26–28] have shown that additional domains beyond occupational factors, (i.e., lifestyle and personal factors), do indeed influence driver physical and mental health, however, we are unsure of the impact of these factors from the driver’s perspective and this study contributes to this body of knowledge.

We therefore, embarked on this qualitative study to explore: What factors support or hinder mental health of Australian truck drivers from their perspective, and what impact do these factors have on their mental health. This research design provides an opportunity to explore the information provided by drivers, thereby adding a richness of understanding for the industry.

## Methods

A Grounded Theory qualitative design was chosen to address the research questions as it gathers in-depth data, provides explicit guidelines for how to conduct the research, how to handle the analytic inquiry and defines the process for constructing middle-level theories from the thematic analysis [29]. Grounded theory provides the opportunity to explore participant ideas and determine an understanding of the interactions between concepts. Interviews were conducted by the first author (PhD, MHScOT) bringing with her over 30 years of experience of working with people in health (occupational therapist) and 10 years’ experience as a qualitative researcher in public health currently as a research fellow. Notes were taken during the interviews.

Relationships between the interviewer and each participant were only established at the time of recruitment, with only basic information given to the participants about the background of the researcher (i.e., a researcher from Monash University). Possible biases and assumptions of the first author were identified as knowledge and expertise in using positive psychology and the science of subjective wellbeing to assist people with refocusing their mindset. These views were not shared with participants, but had the potential to bias the verbal responses and focus of questioning, and analysis of concepts and themes.

Ethical approval was obtained through the Monash University Human Research Ethics Committee (#23388).

### Inclusion criteria

Professional truck drivers ≥ 18 years, self-described as a truck driver, and able to answer questions in conversational English were recruited. Gender was not restricted.

### Recruitment

Truck drivers were purposively recruited using snowball sampling during March – August 2020. We aimed to include a range of drivers e.g. age, gender, geography) although these were not specifically sought.

The three recruitment methods included: First, drivers previously completing a short online health survey [26] who indicated they were interested in providing more in-depth information, were contacted. This in turn spread the word with interested drivers contacting the research team; Second, through a social media campaign on Facebook, Twitter and through the project website (drivinghealth.net); and third, via advertising through study partner networks (emails/flyers) including the Transport Workers’ Union (main Australian transport industry trade union), a large private company employing drivers, and a state-based work health and safety regulator.

Written consent was collected from all participants prior to interviewing with a response rate of 94% of those initially contacted consenting to be interviewed.

### Data collection

Participant demographics captured included gender, age bracket, short- or long-haul driving (< 500kms per day; ≥500kms per day respectively), owner driver or employee driver, state or territories they lived and worked in, size of truck, freight carried, previous workers’ compensation claims, and number of years driving. These categories were determined as important from previous studies in the Driving Health project [26, 27, 30].

Semi-structured questions for drivers [Additional File 1] were developed in consultation with the investigator team, current literature, previous findings from the Driving Health project and known research gaps [31–34]. In line with qualitative methodology, the interview questions were adapted throughout the interviews to explore arising concepts of importance [35].

Interviews were conducted either by phone or video-conferencing (EP), audio recorded and typed verbatim using an external professional service.

### Data analysis

Interview transcripts were analysed using Nvivo software [36] by the first author (EP) in discussion with the senior author (RI – trained physiotherapist and research lead). Initial analysis incorporated open, axial and selective coding phases with a dynamic and iterative thematic analysis to determine concepts and themes [35, 37]. In the axial coding phase, we compared codes with the use of indexing and memo writing to constantly refine and fully capture the diversity of the data as per grounded theory processes [29]. Memos were written within the Nvivo software around the themes beginning to emerge from the researcher’s interpretation, in order to make sense of the data across participants. Some of these examples were titled ‘dishonesty in some companies; frustration around fines; don’t talk about depression; and trapped in a job’. In the final selective phase, we developed the emergent themes from the repeated information and codes, resulting in the presented theory. Examples of codes used were ‘abuse; access to healthy food; alertness; freedom’. In the final selective phase, concepts and themes were derived from the repeated information and codes.

The number of interviews conducted was determined by the methodological principle of saturation [38]. Saturation is reached when no new data arise and any further interviews are unnecessary.

Triangulation [39] was conducted as a means to develop a more robust and richer story through three methods: (1) An independent cross-check of coding (10% of interviews, n = 3) (LW, independent public health researcher not previously involved - see acknowledgments); (2) Comparison of themes with current literature (e.g. Coping methods) completed with input from senior researcher (RI); and (3) Checking of concepts and themes with the *Advisory Group* which included industry and driver representatives.

## Results

A total of 17 interviews were completed (94% male), 16 by phone, and 1 via video-conference, ranging from 28 to 67 min in length with an average of 55 min.

### Demographics

Driver age brackets included 25–34 years (n = 1, 4%), 35–44 (n = 4, 15%), 45–54 (n = 5, 19%), 55–64 (n = 4, 15%), ≥ 65 (n = 3, 12%). Six were short-haul drivers and 11 were long-haul. Thirteen were employee drivers, three were owner drivers, and one was a company owner. Drivers lived and worked across 5 of 6 Australian states and 1 of 2 territories and stated they drove one of following sized trucks; single semitrailer (n = 6), B double (n = 9), double tanker (n = 1), or triple train (n = 1). Varied freight was carried including food and produce, chemicals, building supplies, vehicles or fertilizer. Five drivers discussed having a past workers’ compensation claim (either 2 or 3 claims) some prior to truck driving, some in relation to truck driving. Driving experience ranged from 4 to 58 years with a mean of 25 years. Company size ranged from five vehicles to hundreds (specifics not stated), and working across single-state to multi-state.

### Qualitative analysis

#### Themes and subthemes

Six main themes with two supporting mental health and four posing a risk to driver health were identified. Each theme was complex and categorised into subthemes as discussed below.

### Two themes supporting driver mental health

#### Theme 1: Connections

The primary supportive theme of *connections* extended across three different areas: 1:1) *family connections*; 1:2) *connections with friends/colleagues/mates*; and 1:3) *seeking mental health support from a trained professional*.

### Connections 1:1

#### Family connections

Drivers stated that *family connections* were vital for knowing there was someone to come home to, knowing someone would listen to them when they wanted to chat, and having someone to plan the future with. One driver described this positive impact on his mental health as:

*“They’re [wife and daughters] fantastic, I love them to bits… So that is probably one of the best bits of medicine that I take for my mental health.” Driver 15*

Strategies for staying connected with family ranged from regularly scheduled calls, to having very little sleep to spend time with family (e.g. returning from night shift and staying awake all day), through to sleeping for 24 h on returning home to recover from fatigue and then re-connecting.

### Connections 1.2

#### Friends/colleagues/mates connections

Maintaining *friendships with mates* was often a challenge for many due to the length of time on-the-road and level of fatigue they experienced once back home. However, many discussed the importance of meeting up with mates (close friends) on their days off and how this kept them going and helped them let off steam. Some drivers had mates in the workplace and connected with them over the radio while on the road, particularly at night. Others discussed the importance of connections they had with people on the road, the public and customers they dealt with for pick-ups and deliveries.

*“What I appreciate is the people that you build relationships with, not just in the workplace, but with the customers and clients and so forth, the human interaction.” Driver 05*

### Connections 1:3

#### Trained professional connections

Accessing *trained professionals* was another connection that supported health. Three drivers had sessions with a psychologist, a chaplain, or a trained counsellor. Each of these drivers talked about the value they had received which supported their coping abilities.

*“But I’m very lucky to find this young woman [Psychologist or Psychiatrist - unsure]… I was absolutely stuffed mate, I tried to commit suicide.” Driver 09*

In contrast, three drivers stated they could not access a trained professional when they wanted, either because of the stigma of seeking help from a mental health professional or because irregular shifts made it difficult to access a health service at a suitable time.

#### Theme 2: Current coping methods

The second supportive theme of *Coping methods* describes the strategies drivers engaged in, to cope with the challenges of isolation and tedium when on the road alone. This theme incorporated five subthemes: 2:1 *appreciating the moment* (engaging senses and noticing the beauty in the moment); 2:2 *practising gratitude* (showing heartfelt appreciation); 2.3 having an *optimistic mindset* (deep seated belief that things will work out); 2.4 having *mental toughness* (a developed psychological edge to cope); and 2:5 using *internal versus external locus of control (LOC)* (being driven by internal motivations and desires and not leaving things to chance).

### Coping Methods 2:1 and 2:2

#### Appreciating the moment and practising gratitude

Most talked about how they were able to *appreciate the moment* of the beauty they saw while on the road, the places they got to see, feel and experience and the events of nature they were thankful to experience in the moment *(practising gratitude)*.

*“The things I really like [is] the freedom, the freedom of doing what I want when I want. And to be one with the elements...” Driver 06*

They shared how they were thankful for the opportunities to provide for their family, be in the truck and have alone time, and also be able to experience the wonders of nature. These acts of *appreciating the moment* and *practicing gratitude* made things much more bearable and offset, in some way, the isolation they experienced on long-haul trips.

### Coping Method 2:3 and 2:4

#### Optimistic mindset and mental toughness

The degree to which a driver defined themselves as coping (or not), was identified by the researchers as being linked to their *mindset* (optimistic or pessimistic) and level of *mental toughness* they possessed. That is, those with a more *optimistic mindset*, where they absolutely believed things would somehow work out in the future, [40] and those with *mental toughness*, (“having the natural or developed psychological edge that enables you to, generally, cope better than your opponents with the many demands” [41]), appeared to cope better with the separation and isolation from family, and made choices that supported their general and mental health.

*“If you’re having an emotional day you’re in a controlled environment, where you can deal with it, whether you ignore it and distract yourself or you find something on the internet, a podcast or something where you can self-counsel.” Driver 10*

*Mental toughness* for some, was paramount where drivers recognized their level of resilience (“adaptation in the face of adversity…” [42]) or degree of ‘GRIT’ (“passion and sustained perseverance applied toward long-term achievement” [43]) they possessed. *Mental toughness* is a skill that can be honed and developed over time and is not about adopting a “bravado attitude” known as a “temporary bluff” [43], therefore drivers that displayed these characteristics found it easier to cope on the road, than others.

### Coping Method 2:5

#### Internal versus external locus of control

Some drivers displayed high levels of *Internal LOC* where they described how they alone were responsible for their responses to stressful situations. *Internal LOC* is the degree individuals perceive an outcome is created by their own actions in contrast with *external LOC* where individuals perceive outcomes governed by luck or factors beyond their control [44]. This was in contrast to others who struggled more and felt controlled by ‘them’ (the bosses, environment or industry regulations). Research has shown that those with higher *internal LOC* have higher levels of work satisfaction [45] and better health outcomes [44, 46], whereas those with higher *external LOC* tend to experience higher levels of stress and depression [47], more anxiety, learned helplessness [48], and display little faith in themselves [49].

*“Mum and Dad said to me, years ago, ‘you make your own way in this world. You’ve got to learn to run your own race’. If you’re going to follow the sheep the view will never change”. Driver 10*

Drivers appearing to cope better with the excessive demands of driving embraced the principles of recognizing what they could control (*internal LOC e.g., their daily mood and responses*) and tended to let go of the things they could not (e.g. the scheduling of shifts, the traffic). They did not blame external factors or people within the industry, and tended to just get on with the job each day. They also demonstrated an ability to instigate higher levels of willpower to make healthier food and exercise choices during their day.

### Four themes presenting a risk to mental health

#### Theme 3: Compromised support systems

Even though *connections* supported driver health, many drivers identified how their support systems were also compromised. These appeared to arise from long separations from family, the demands of the job, lack of energy when home, and often led to relationship breakdown or divorce. This theme had three sub-themes: 3:1 *separation*; 3:2 *relationship breakdown*; and 3.3 *stigma*.

### Compromised support systems 3:1

#### Separation from family

For drivers, the *separation* from family was compounded with the guilt they felt when away from home and being unable to attend family events or sort things at home when needed. Drivers shared about the many milestones, birthdays, school or sporting events they missed with their children, and how this put additional strain on the couple and family relationships and negatively impacted their support systems.

*“My daughter takes it hard because whenever [I] go away she gets really upset… And [my wife] freaks out when there are noises around the house,… that’s quite hard to take.” Driver 08*

The separation from their family also took its toll on their mental health, as being alone for extended times was when they experienced escalating negative thoughts.

*“Where you start getting some really depressing thought… being there by yourself it escalates and multiplies and sometimes can get really bad.” Driver 16*

### Compromised support systems 3:2

#### Relationship breakdown

Several recognized that they tended to offload and transfer their stress onto their partners rather than seek professional help, in a bid to get some level of acceptable support. This put additional pressure on already fragile relationships often leading to *relationship breakdown*.

### Compromised support systems 3:3

#### Stigma

Most drivers discussed their reticence to access formal professional support due to the *stigma* attached and their belief that the services did not understand truckies and therefore could not help. They also talked about their *bravado*, *male mentality*, and stated they were tough and did not need help.

*“Like with most male truck drivers, we do not communicate with a lot of people. We’re the tough guys...” Driver 05*

#### Theme 4: Unrealistic demands

Drivers stated many instances of *unrealistic demands* which fell into two sub-themes: 4:1 *lack of control in their role*; and 4:2 *the flow on effect* of ‘the system’ and external environmental factors.

### Unrealistic demands 4:1

#### Lack of Control

Drivers frequently identified areas of *lack of control* discussing how they had to stick rigidly to the regulations even when they were impractical. This was usually connected with roadworks or loading delays or other external factors that stopped them meeting delivery schedules. They were fearful about regulation breaking to meet delivery schedules, as even a small unintentional diversion could cost them a fine of 1-2-week’s wages. This increased anxiety and the financial impact of not meeting work demands, even further.

Examples of factors that were outside the driver’s control were stories of driving an extra 23 kms (over their allowed daily limit) because the parking bay was full of caravans. Another discussed the lack of control over when they stopped or rested and were unable to stop despite feeling fatigued, because the logbook stated it was ‘driving time’. They talked about feeling weak or powerless to discuss any schedule deviations with their bosses or manage their time, energy or fatigue.

*“I’d be driving and I knew I should have been taking a break… I didn’t want to be like a sissy and ring up the boss and say… I’m not going to make it.” Driver 02*

Many discussed the lack of control they had over their shift selection and the uncertainty of where they would be tomorrow or next week. They also lamented the in-flexibility of management to approve leave (even for important events), when requested.

### Unrealistic demands 4:2

#### Flow on Effect

The *flow on effect* of the systems, shift allocation, unforeseen delays and waiting times, compounded throughout the week, which drivers felt exacerbated unrealistic demands even further. This often flowed onto missing delivery timelines and not being able to make it home when originally planned, creating even more separation from family.

*“I’ve got to put the waiting time in my work diary as work time [unpaid]… which means I’m going to run out of hours, two hours prior to getting back home”. Driver 06*

The flow on effect of external factors such as weather, traffic, road-works, lack of on road facilities, and limited parking bays were all identified as compounding unrealistic demands that negatively impacted driver health. The lack of supportive infrastructure providing essential on-the-road facilities (e.g., toilets, clean showers) were constant complaints. One driver stated he felt *“like an animal”*, toileting on the side of the road. All of these factors further eroded energy, self-worth and created more fatigue and at times, exacerbated hazardous driving conditions.

*“So you’re right at the edge of your fatigue zone… just struggling to stay awake and there’s nowhere to pull over.” Driver 02*

Drivers offered very few suggestions or solutions to these demands except to change the unrealistic nature of them into one that was more realistic for their body and time.

#### Theme 5: Financial pressures

Financial pressures were discussed by drivers within two subthemes: 5:1 *unpaid waiting time* and the impact on delivery completions each week; and 5:2 the *competitiveness of the market* where companies were underbidding to secure contracts.

### Financial pressures 5:1

#### Waiting Time

No-one was paid for their *waiting time*. Drivers indicated how waiting times encroached into their logbook daily as technically they were “resting” however they still needed to be alert in their truck, gradually moving up the line in preparation to load/unload. This increased fatigue levels to the extent where the driver was often unable to fulfil delivery requirements, therefore unable to collect the next load, which in turn impacted their take-home pay.

*“[There’s a problem when]… you’ve got a 10-hour drive in front of you… some places you can wait three hours...” Driver 04*

### Financial pressures 5:2

#### Competitiveness of the market

Drivers who had been in the industry > 5 years stated how they noticed increasing *financial pressures* and a reduction in their take-home pay over time. Quotation of jobs were more competitive, and some companies were undercutting others by servicing the trucks with three immigrant drivers (temporary visa/casual workers) which meant cheaper salaries (e.g., no sick leave paid). This kept the truck on the road 24/7 with no road-side stops needed and meant undercutting single and smaller driver operations. Truck rental payments and overheads were also increasing while the profitability of loads was decreasing, placing additional financial pressures on both drivers and owners.

*“Look there’s been lots of stress. A lot of it is to do with when you don’t have much sleep or you’ve got bills that you’ve got to pay or you don’t have the money to pay the bills.” Driver 03*

#### Theme 6: Lack of respect or recognition

Lack of respect or recognition was identified by drivers and experienced across two different subthemes: 6:1 *managers/company*; and 6:2 the *public*.

### Lack of respect or recognition 6:1

#### Managers/company

Drivers shared experiences of *lack of respect from managers* and how they felt unappreciated and often experienced verbal abuse throughout their day.

*“Management, the biggest problem we have as a truck driver, we get spoken to like shit. We are the bottom of the chain.” Driver 03*.

One described his experience of witnessing racism and discrimination within his workplace, where management thought themselves as more important.

*“… just in a general racism way because some people think they’re better than others and you can see sometimes that that is directed…” Driver 04*.

### Lack of respect or recognition 6:2

#### The public

Several mentioned the *lack of respect from the public* including receiving bad press from media and police. They often discussed how they experienced blame for traffic crashes even when they were first on the scene helping others out. Some shared how they were verbally abused for no apparent reason by motorists or cyclists.

*“He just lunged straight into attack mode and because we were moving so slowly he sat beside the truck yelling abuse at me.” Driver 04*.

When asked why they remain in a job where they are not respected or appreciated, many replied, “what else would I do?”, exhibiting again a lack of control they felt they had over their own lives and career.

## Interactions across themes

### The model of work and coping factors impacting truck drivers’ mental health

**Fig. 1:**
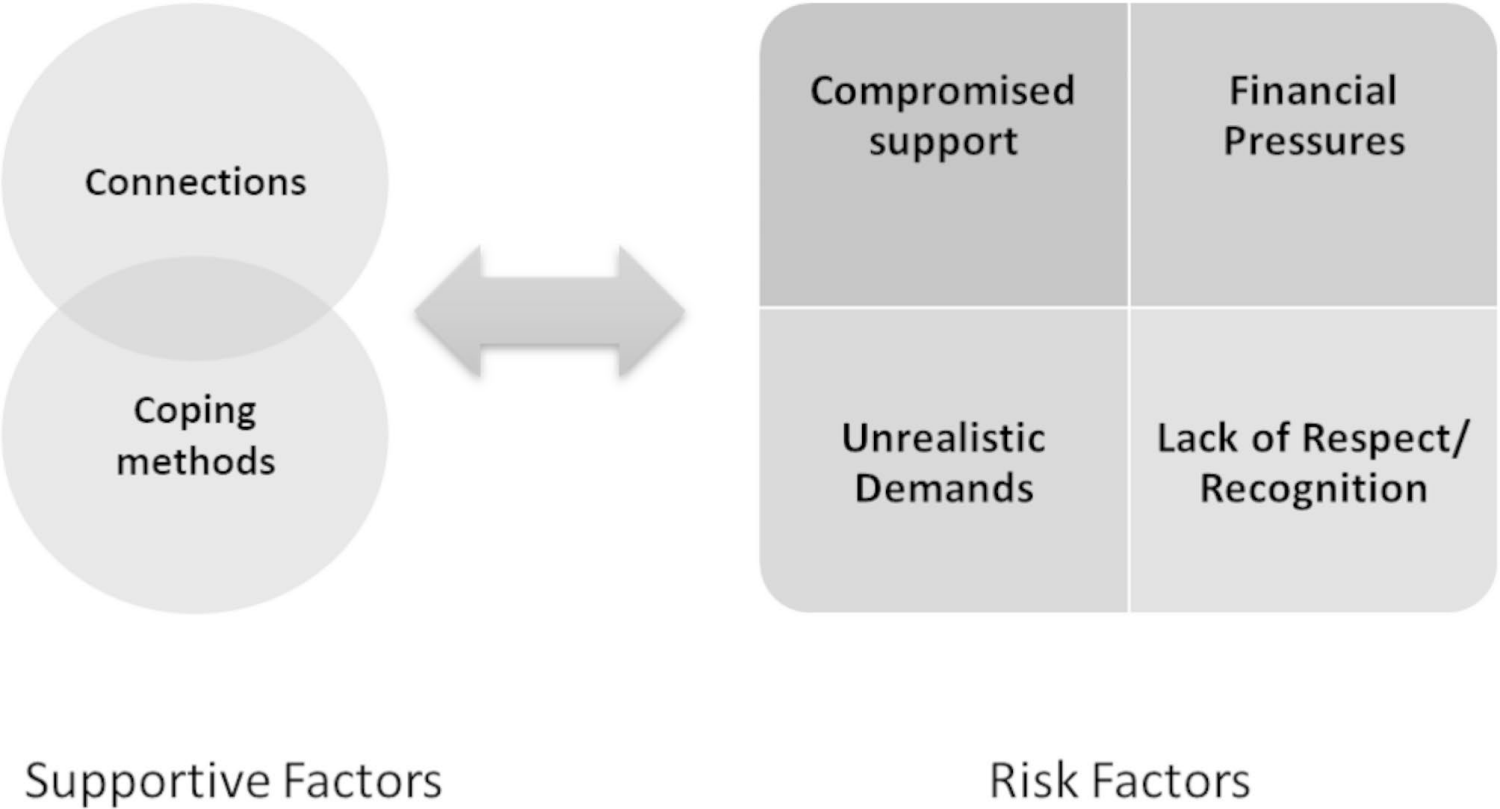
Truck driver work and coping factors impacting mental health

The 6 themes were all interwoven, including those that supported and those that posed a risk to health. The two supportive factors (*Connections* and *Coping methods*) appeared to have some overlapping elements. For example, the abilities of the driver to find a way to mitigate the potential barriers to maintain strong connections with family or friends, overlapped with their coping methods and abilities to find a way to make it happen. The four themes posing a risk to mental health (*Compromised Support*, *Unrealistic Demands*, *Financial Pressures*, and *Lack of Respect or Recognition*) appeared to be independent factors (Fig. 1).

An interesting interaction was noted between *Connections* and *Compromised support* where the level of support drivers received from family members and mates was paramount to their health but often the hardest to maintain due to *unrealistic job demands* (risk factor). Accessing professional support was juxtaposed for many to the stigma around getting support for mental health even though some drivers had experienced the value of this support, and others stated they (or their workmates) would never access this possibility.

The interactions between the themes posing a risk to health were difficult to untangle. *Unrealistic demands* predominantly from the workplace was also incorporated with unrealistic demands from family which for some, led to relationship breakdown. *Lack of recognition* and *Financial pressures* were linked as drivers stated that if they were more respected, then there would be higher rates of remuneration and less ‘cut throat’ costing practices in place.

Current *Coping methods* and *Compromised support* interacted as many enlisted their optimistic coping methods to deal with relationship breakdown. *Coping methods* were also enlisted to get through the *Unrealistic Demands*, *Financial pressures* and *Lack of respect or recognition*. In addition, it is worth noting that the themes supporting health are more likely to be within the drivers control, but the negative factors have a strong external focus.

Despite the hardships discussed by all participants, there were also intermittent rays of hope. Some had experienced being treated with respect by management and the public, some applauded the new initiatives some companies instigated to look after their drivers, and others commended their long-suffering partners for sticking by them no matter what. Overall, the trucking industry appears to be inconsistent in the treatment of drivers with large diversity of approaches across states and companies without any clear solutions presented.

## Discussion

This study aimed to identify and describe work and coping factors that either supported or put at risk the mental health of truck drivers from their perspective. Six themes emerged, with two that supported health (*Connections; Current coping methods*) and four that posed a risk to driver health (*Compromised support systems*; *Unrealistic demands; Financial pressures; and Lack of respect or recognition*).

When looking at the sub-themes supporting health, there was a spread across the continuum of being driven by an *internal LOC* or an *external LOC*. In line with current research, drivers who displayed an internal LOC (identified by the researcher from the language used in responses to interview questions) appeared to take more responsibility for their health. This reflects literature regarding the importance of having internal LOC as a human psychological driver of mental health [50, 51]. The work of Gore et al., also reports that those with higher internal LOC make better health choices around exercise and those with higher external LOC have ‘positively predicted unhealthy behaviours’ e.g., eat more junk food, have higher levels of substance abuse [52]. One way to move from a more external to internal LOC is teaching critical and creative thinking skills which has been shown to improve psychological well-being [53]. However, this type of research has not been conducted with truck drivers to ascertain if improving coping strategies and moving from a more external to internal LOC, would improve their mental health status. Therefore, locus of control is an important area to consider in future research to ascertain the possible impact on driver mental health.

The interactions across each of the themes identify the complex nature of the transport industry. The level of social support a driver has, how they maintain and grow their connections and relationships, the coping methods they possess, the level of internal LOC they have, and their beliefs around seeking professional help, create an interplay of factors. We know that the quality and quantity of social relationships is important for mental and physical health [17], however this is an area that is compromised among truck drivers due to the isolated and lonely nature of their role [21, 27, 54]. Drivers who had higher levels of these coping strategies (as determined by the researcher) or stated they sought professional help were able to mitigate some of the health risks. Furthermore, we know that health seeking behaviours in the driving industry are compromised for multiple reasons e.g. the stigma attached to seeking help, barriers around accessibility to attend appointments, lack of control over shifts [21, 55] and that drivers do not always receive evidence-based care [56]. We also know that truck drivers tend to downplay their health risks and that there are environmental and attitudinal barriers preventing them from following through with their intentions to make lifestyle changes [22, 57]. Previous literature has also highlighted that the risk factor sub-themes identified in this study can lead to varied medical conditions, increased crashes, and poor mental health and need to be mitigated where possible [4, 28, 54]. These risks are also echoed across other occupational groups and pose ongoing challenges for creating effective interventions to reduce years of time lost, lost productivity and future health costs for treating mental ill-health [14, 25]. Many of the environmental and risk factors are beyond the control of the drivers and highlights the importance of a multi-faceted approach to strengthen the skills that the driver can control including coping skills, in conjunction with addressing the part each stakeholder plays [30, 31, 58].

There are no simple solutions to address the complex interplay of factors experienced by drivers on a daily basis. For example, solely addressing and enhancing coping mechanisms of drivers is unlikely to create long-term improvement in mental health if other factors in the industry, environment and the public, are not simultaneously addressed. Previous research has identified that in some cohorts, improving coping strategies can be taught [59], and indeed have a positive impact on reducing pain and anxiety in adults with arthritis [60] however, this has not yet been explored in the trucking industry. Additionally, resilience interventions that focus on building “protective factors” (e.g., a sense of belonging, accessing social support, self and affect regulation, cognitive restructuring skills, self-esteem, and self-worth) have been found to be effective at promoting positive mental health outcomes in high risk populations [61]. Evidence also shows that resilience interventions can prevent and address mental health disorders, making them a valuable tool for promoting positive outcomes [62, 63]. An RCT exploring Resilience@Work Mindfulness programs with first responders identified an improvement in adaptive resilience [62]. Another example is the RAP-A, a school-based resilience intervention with established efficacy in promoting resilience and growth, reducing mental health symptomatology and preventing the development of mental health disorders [63, 64]. This program has been adapted to high-risk occupations including police officers [63] and could be tailored to the transportation industry.

This study shows that whatever future solutions are offered, the interventions need to: (1) target all levels of the industry and not just the driver; and (2) constantly consider the flow on effects of any change, for the driver. For example, when changing the logbook regulations where drivers are no longer able to control their sleep/wake times even when feeling fatigued, then recognition of the impact on driver health needs to occur. This study indicates that a wide-reaching systematic approach across all stakeholders (companies, regulators, unions, general public, drivers, and family members) is required if we are to advance the mental health of Australian truck drivers. Furthermore, alongside the industry addressing these issues simultaneously, there is also a need for interventions targeted at drivers to include a component of counselling/training to increase the driver’s internal LOC, which in turn will strengthen their abilities to cope with the realities of the job.

## Strengths and limitations

A key strength of this study lies in the diversity of drivers interviewed providing insight into drivers living and working across Australian states, with varied age ranges, and a breadth of experience. Another strength is the use of a grounded theory and inductive analysis approach, which has enabled us to identify the work and coping factors and their impact. Additionally, triangulation of the results also confirmed the robustness of the findings.

A study limitation was the impact of the challenges of the COVID-19 pandemic outbreak in 2020 which impacted speed of recruitment, and the possibility of in-person interviews. Additionally, convenience and snowball sampling may have resulted in an atypical and biased sample of drivers of those who were keen to talk about industry problems from their perspective. As per the principles of qualitative research and the numbers of participants involved, results cannot be generalised to other populations or experiences. Furthermore, even though the sample included one female driver’s experience (reflective of the male/female statistics of Australian drivers), this may not reflect the experience of other women in the industry.

## Conclusion

This qualitative study has enabled us to understand the factors that supported or posed a risk to the mental health of truck drivers. The strength of *connections* and number of *coping strategies* each driver has appears to enhance mental health, whereas *compromised connections*, *unrealistic demands*, *financial pressures* and *lack of respect or recognition*, pose risks to driver health. Another consideration for driver health emerged in relation to whether the driver had an internal or external locus of control. Possessing an internal LOC appeared to be one of the attributes that can overcome the lack of control of many situations experienced daily and from companies, regulators, the public or even the environment.

Although intervention strategies targeting drivers are necessary, one cannot take a “driver only approach” as systemic improvement is clearly needed. Only concentrating on drivers may even hamper system-wide approaches to reducing drivers’ mental and physical health risks at work. Interventions to address the complex phenomena of driver mental health need to be designed with input from all industry stakeholders, including family members, and be delivered at multiple levels within the transport industry in order to be effective.

## Electronic supplementary material

Below is the link to the electronic supplementary material.

Supplementary Material 1

## Data Availability

The datasets generated and/or analysed during the current study are not publicly available due to being interview transcripts and may identify the participant their workplace and contacts but are available from the corresponding author on reasonable request.
