# Supplementary material for: Impact of work and coping factors on mental health: Australian truck drivers’ perspective
Source: BMC Public Health. 2023 Jun 6;23:1090. doi: 10.1186/s12889-023-15877-4 (PMC10242603; doi:10.1186/s12889-023-15877-4)
Supplement: Supplementary file 1 — Supplementary Material 1 [file 12889_2023_15877_MOESM1_ESM.docx]

**Additional File 1.**

**Questions for semi-structured interviews (process over time – for grounded theory design) DRIVER**

1. What areas of truck driving do you like/ dislike?
2. How would you describe your own health right now?
   1. How has this changed over the time you have been driving?
3. What factors improve or impair your health? E.g. Work factors, experience, training, health conditions, food intake, movement/exercise, equipment used -loading, unloading, the rigs, being on the road alone
   1. How do they improve or impair your health?
   2. What impact does driving have on your sleep or stress?
   3. How do you manage the fatigue or stress?
   4. When or how do you access doctors/ or health clinics if you need them?
4. Have you ever made previous attempts to improve your health?
   1. What worked?
   2. What didn’t work?
5. What would help support you and your health in the future?
   1. From your employer
   2. from yourself
   3. from your family
   4. from GPs or health clinics
6. What wouldn’t work?
   1. Information
   2. Incentives
   3. Digital aps/ devices (e.g. fitbit, pedometer)
   4. Coach
   5. Policies
   6. Programs
7. Have you ever had a truckie mate that has struggled with depression or stress?
   1. How did this affect you?
   2. What are your thoughts around driving and stress? (physical, emotional, financial)
8. How does driving affect your family life?
   1. Would it be possible to speak with someone from your family to also get their perspective?
9. How do you get on with the people you work with/ for? (in the truck, loading, paperwork processing etc)
   1. How has this changed over time?
   2. How does this improve or impair your health?

**Demographics:**

- Age bracket
- Long-haul/ short-haul?
- Type of rig/ loads
- Any previous claims? – What for? – Musculoskeletal, other trauma, fractures, neurological (including hearing), mental ill health, other diseases
- Owner operator/ employed – how big is the company – how many drivers employed?
- How long have you been truck driving?
